# Evaluating blue light impact on reconstructed human epidermis using laser speckle imaging

**DOI:** 10.1117/1.JBO.30.5.056001

**Published:** 2025-05-07

**Authors:** Léa Habib, Léa Abi Nassif, Marie Abboud, Rime Michael-Jubeli, Ali Tfayli, Roger Lteif

**Affiliations:** a Université Saint Joseph, Faculté des Sciences, Laboratoire d’étude cinétique en milieu hétérogène, Beirut, Lebanon; b Université Paris-Saclay, Faculté de pharmacie, Unité Universitaire Interdisciplinaire Lip(Sys), Lipides, Systèmes analytiques et biologiques, Orsay, France; c Université Saint Joseph, Faculté des sciences, Physics Department, UR TVA, Beirut, Lebanon

**Keywords:** reconstructed human epidermis, epidermal models, blue light, visible light, speckle, barrier function

## Abstract

**Significance:**

Blue light exposure is ubiquitous in modern life, raising concerns about its potential impact on skin health.

**Aim:**

We aim to explore the effects of blue light on the reconstructed human epidermis (RHE) using the speckle analysis technique.

**Approach:**

RHE samples were irradiated with controlled doses of blue light (415 and 455 nm) at defined stages of their maturation. Following irradiation, speckle analysis was performed to assess the impact of blue light on the skin barrier.

**Results:**

Our results demonstrate that blue light irradiation significantly alters the scattering properties of RHE. Both wavelengths induced changes in the degree of linear polarization and speckle grain size, indicating disruptions in the skin barrier’s structure and organization. The effects were found to be wavelength-dependent, with 455 nm irradiation showing more pronounced changes.

**Conclusions:**

Speckle imaging allowed detection of changes in the scattering properties of RHE. Findings suggest that blue light exposure can influence skin barrier function and may have implications for skin health and related conditions.

## 1 Introduction

Since their inception on the planet Earth, humans have been exposed to different intensities of a broad spectrum of electromagnetic radiation, including both visible and nonvisible wavelengths.^1^ The human skin serves as the primary defensive barrier against several environmental factors, including these radiations.^2^

Blue light (400 to 500 nm), a component of the visible light spectrum, has gained significant attention due to its increasing prevalence in modern lifestyles. With the widespread use of electronic devices and artificial lighting, individuals are exposed to blue light for extended periods daily. Although most research has focused on blue light’s impact on the eyes, the understanding of its effects on the skin, which contains similar photoreceptors, remains limited.^3^^–^^5^

Previous studies have primarily focused on the impact of ultraviolet (UV) radiation on the skin, highlighting its role in causing sunburn, premature aging, and skin cancer.^6^ However, recent research has revealed that blue light can also induce oxidative stress, inflammation, and DNA damage in skin cells, leading to various skin concerns.^3^^,^^7^^–^^9^

The shift toward reducing animal testing has driven the development and validation of several *in vitro* skin models. These include full-thickness human skin equivalents (HSEs),^10^ which incorporate both dermis and epidermis layers, and three-dimensional bioprinted skin constructs.^11^ In addition, human skin explants maintain the complete skin architecture and appendages.^12^ Other established models involve reconstructed human epidermis (RHE) models, which provide a valuable *in vitro* platform for investigating the effects of various stimuli,^13^^–^^16^ including phototoxicity,^17^^,^^18^ on skin physiology. Each model presents unique advantages: HSEs provide a more physiologically relevant representation of dermal–epidermal interactions,^19^ whereas RHE models are particularly suited for investigating barrier function.^16^ Given that these models mimic the key structural and functional characteristics of the human epidermis, they serve as a reliable platform for controlled experimentation and the assessment of specific cellular responses.^20^^,^^21^ Over the years, these models have shown notable improvement in reproducibility and reliability.^22^

Laser speckle imaging, a noninvasive optical technique widely employed in fields such as biomedicine and materials science, complements this approach effectively.^23^^–^^27^ By analyzing the interference pattern resulting from laser light scattered by an illuminated sample, speckle analysis provides insights into its microstructure and dynamics. Hence, once speckle analysis is applied to RHE models,^28^ we can gain valuable information about the structural changes induced by blue light exposure.

This study aims to investigate the impact of blue light exposure on the reconstructed human epidermis and to assess the effects of this light on the skin barrier.

## 2 Materials and Methods

### 2.1 Reconstructed Human Epidermis SkinEthic™

This study employed three-dimensional reconstructed RHE models from Episkin^®^ (L’Oréal, Clichy, France). These SkinEthic™ RHE models are a well-established tool for replicating the human epidermis. They effectively replicate the natural key epidermal layers by growing normal human keratinocytes on an inert polycarbonate filter at the air–liquid interface. This includes the organized basal, spinous, and granular layers, topped by a multilayered stratum corneum.^29^ RHE models were obtained on day 11 of the differentiation process (D11). To reach a more mature epidermal state, the models were further cultured in fresh growth medium for an additional 3 days, reaching a differentiation stage corresponding to day 14 (D14). This culture was performed in 6-well plates with 2 mL of growth medium per well under standard cell culture conditions (37°C, 5% ${\mathrm{CO}}_{2}$). Following this incubation period, RHE models were irradiated by blue light according to various experimental conditions.

### 2.2 Blue Light Exposure

#### 2.1.1 Light sources

This study employed two light-emitting diodes (LED) from Thorlabs Inc. (Newton, New Jersey, United States) that differed in their emission peak, with one emitting primarily at 415 nm (considered blue-violet) and the other at 455 nm (turquoise blue). The average irradiance (light intensity delivered) of the 415 nm LED was $∼35\pm 5\;\mathrm{mW}/{\mathrm{cm}}^{2}$. Similarly, the 455 nm LED delivered an average irradiance of $29\pm 3\;\mathrm{mW}/{\mathrm{cm}}^{2}$.

The exposure time for each RHE model was adjusted to deliver a consistent dose of $50\;J/{\mathrm{cm}}^{2}$. This resulted in an average irradiation time of 20±0.5  minutes for the 415 nm LED and 26±0.5  minutes for the 455 nm LED. These times were not obtained by dividing the total dose by the mean irradiance across all experiments but were instead calculated as the average of the irradiation times determined individually for each irradiation, based on the irradiance measured for that specific session. This specific energy dose was selected as it approximates the amount of blue light exposure one would receive from 1.5 h of sunlight during summer.^30^^,^^31^

#### 2.2.2 Experimental setup

**Fig. 1:**
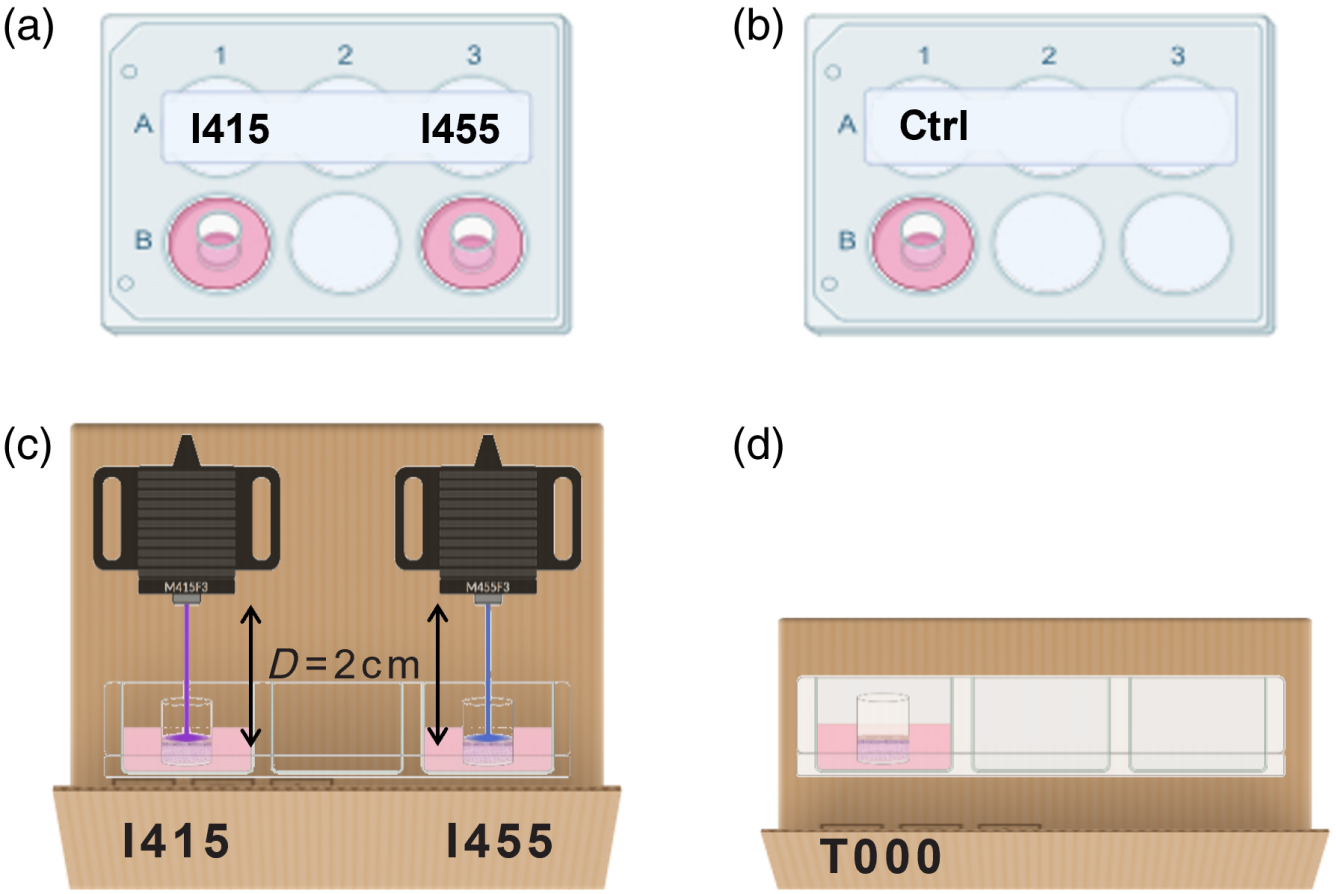
Experimental setup for RHE model irradiation. (a) 6-Well plate containing RHE models designated for specific wavelengths: I415 for 415 nm and I455 for 455 nm exposure, with each model placed in separate wells to avoid cross-contamination. (b) Separate 6-well plate containing unirradiated control models (Ctrl). (c) Irradiation compartment with the LEDs positioned at a distance of D=2  cm from the RHE models and (d) control compartment.

Individual SkinEthic™ RHE models were assigned designations based on the light they would receive: I415 for 415 nm exposure and I455 for 455 nm exposure. These models were placed in separate wells of a 6-well plate to prevent cross-contamination during irradiation [see Fig. 1(a)]. The plate was sealed to maintain sterility throughout the experiment.

An unirradiated control group (Ctrl) was prepared using a separate plate [Fig. 1(b)]. This control mimicked the environmental conditions experienced by the irradiated samples, excluding the exposure to blue light [Fig. 1(d)].

To ensure consistent light distribution across the RHE surfaces, the entire experimental setup was enclosed within a light-tight chamber. In addition, the LEDs were positioned at an optimal distance of 2 cm from the RHE models [Fig. 1(c)]. The irradiation protocol involved exposing the models every 24 h for several consecutive days (3 to 6 days) during their maturation process.

The experimental design encompassed a range of irradiation conditions, as detailed in Table 1. For each condition, there was one sample subjected to 415 nm light irradiation (designated as I415), another exposed to 455 nm light (labeled I455), and a third serving as the control group (denoted Ctrl), which remained unexposed to any irradiation.

**Table 1:** Experimental conditions for each sample.

Sample name	Irradiation repetition	Total dose ($J/{\mathrm{cm}}^{2}$)	RHE maturation day	Measurement
R3_D17	3	150	17	Immediately after the last exposure
R4_D18	4	200	18	
R6_D20	6	300	20	
R3_D18	3	150	18	24 h post-last exposure

### 2.3 Speckle Image Acquisition

A linearly polarized He–Ne laser beam (λ=632.8  nm, 7 mW) was directed onto the RHE insert. Backscattered light at a θ=20  deg angle was captured by a high-speed CMOS camera (Motion-Blitz EoSens Mini 1, pixel size 14  μm×14  μm) positioned at a distance D=20  cm from the sample. An analyzer was used to control the polarization of the detected backscattered light.

Speckle images (400×400  pixels) were collected under parallel polarization conditions and were subsequently analyzed to extract relevant speckle parameters.^28^

The speckle pattern produced by each illuminated sample is influenced by the medium’s properties, including particle size and diffusion coefficient.^32^ When particles are significantly larger than the optical wavelength, diffusion primarily occurs in the forward direction. However, for smaller particles, light is scattered uniformly in all directions.

Speckle images reveal important information about the polarization properties of scattered light. In fact, the scattering properties of each illuminated sample are influenced by its unique particle size distribution, which affects both the diffusion regime and the polarization of the scattered photons. By quantifying the polarization characteristics of the backscattered light, particularly through the degree of light polarization (DOP), one can access information about the size of scatterers. DOP is calculated using Eq. (1)

$$\mathrm{DOP}=\frac{I_{//}-I_{⊥}}{I_{//}+I_{⊥}},$$ (1)

where $I_{//}$ and $I_{⊥}$ represent the average intensities measured when the polarization of scattered light is parallel and perpendicular, respectively, to the plane of polarization of the incident light.^32^

In this study, we specifically analyze the DOP for linear polarization (${\mathrm{DOP}}_{L}$), which refers to the fraction of backscattered light that retains linear polarization. Although linear polarization can exist in any direction within the xy plane, our setup captures the horizontal (parallel) and vertical (perpendicular) components, from which we compute ${\mathrm{DOP}}_{L}$. This allows for the evaluation of the dominant characteristics of the scatterers present in the diffusing medium and the detection of relative changes in their size.^24^^,^^32^^,^^33^

The horizontal speckle grain size was estimated using the normalized autocorrelation function of the speckle intensity pattern. This function, obtained from the camera’s observation plane, provides a measure of the average speckle grain width.^34^ Calculated using the Wiener–Khintchine theorem, the normalized autocorrelation function is defined by Eq. (2), where FT represents the Fourier Transform and ⟨⟩ denotes spatial averaging. The horizontal dimension of the speckle grain, denoted as dx, was determined as the full width at half maximum (FWHM) of the horizontal profile of the autocorrelation function.^34^

$$c_{I}(x,y)=\frac{FT^{-1}[|FT[I(x,y)]{|}^{2}]-I(x,y{)}^{2}}{I^{2}(x,y)-I(x,y{)}^{2}}.$$ (2)

Speckle grain size provides information about the size of scatterers with respect to the optical wavelength.^33^ In addition, it can offer insights into the surface state or texture of the illuminated diffusing medium.^26^^,^^27^

## 3 Results

### 3.1 Reassessment of Control Groups: Impact of Experimental Conditions

To accurately assess the influence of irradiation on sample response, a control group was essential. Although a standard control within the incubator was initially considered, the perturbation caused by removing samples from the incubator for irradiation purposes necessitated a more appropriate comparison.

**Fig. 2:**
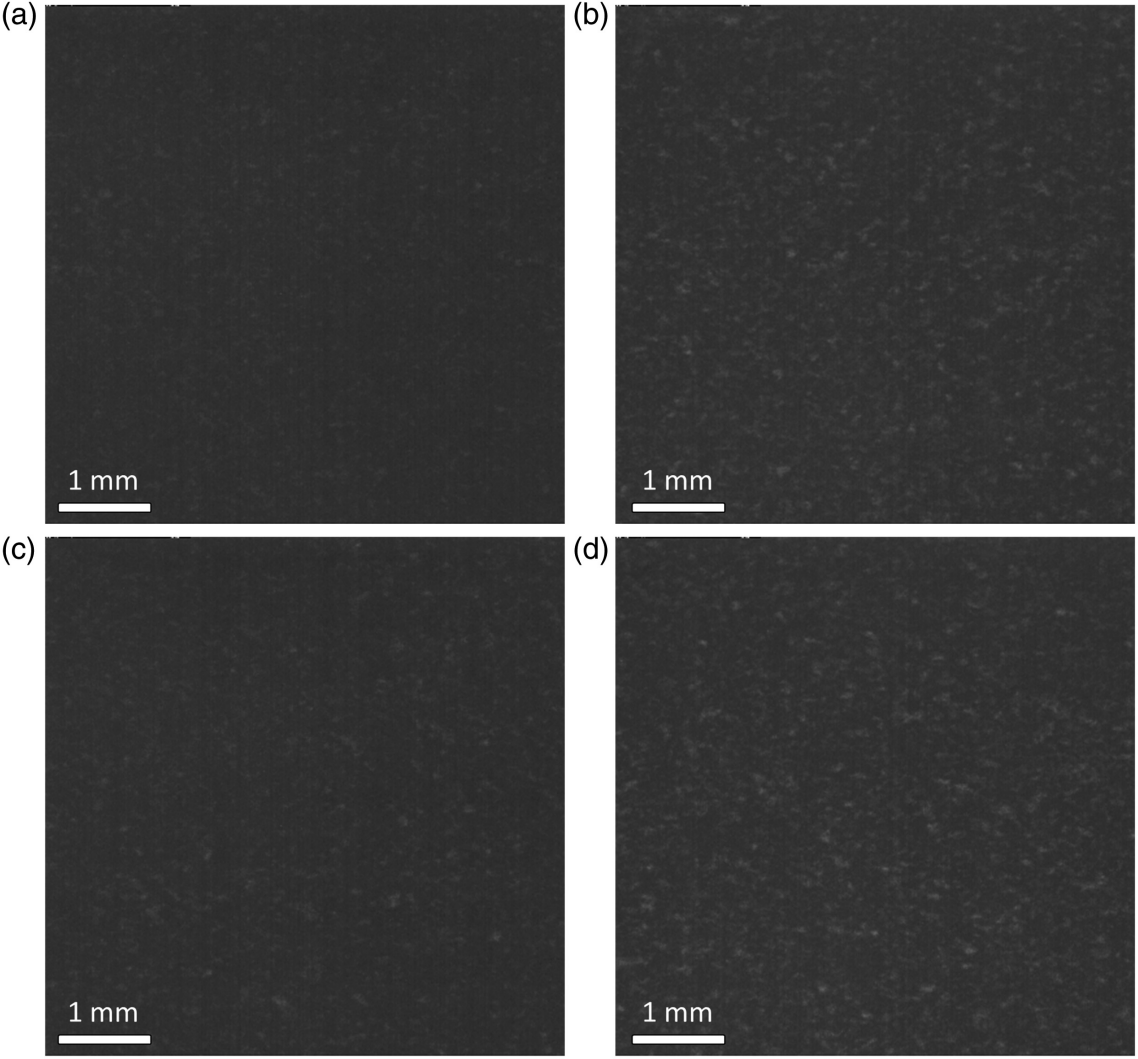
Speckles image recorded using linear parallel polarized detection of the RHE standard controls (a) D17 and (b) D20 and groups with repeated incubator removals (c) R3_D17 and (d) R6_D20. RHE, reconstructed human epidermis.

To investigate the impact of removal from the incubator on RHE properties, we compared speckle (Fig. 2) parameters between standard controls (D17 and D20) and groups subjected to removal from the incubator three times (R3_D17) and six times (R6_D20).

**Fig. 3:**
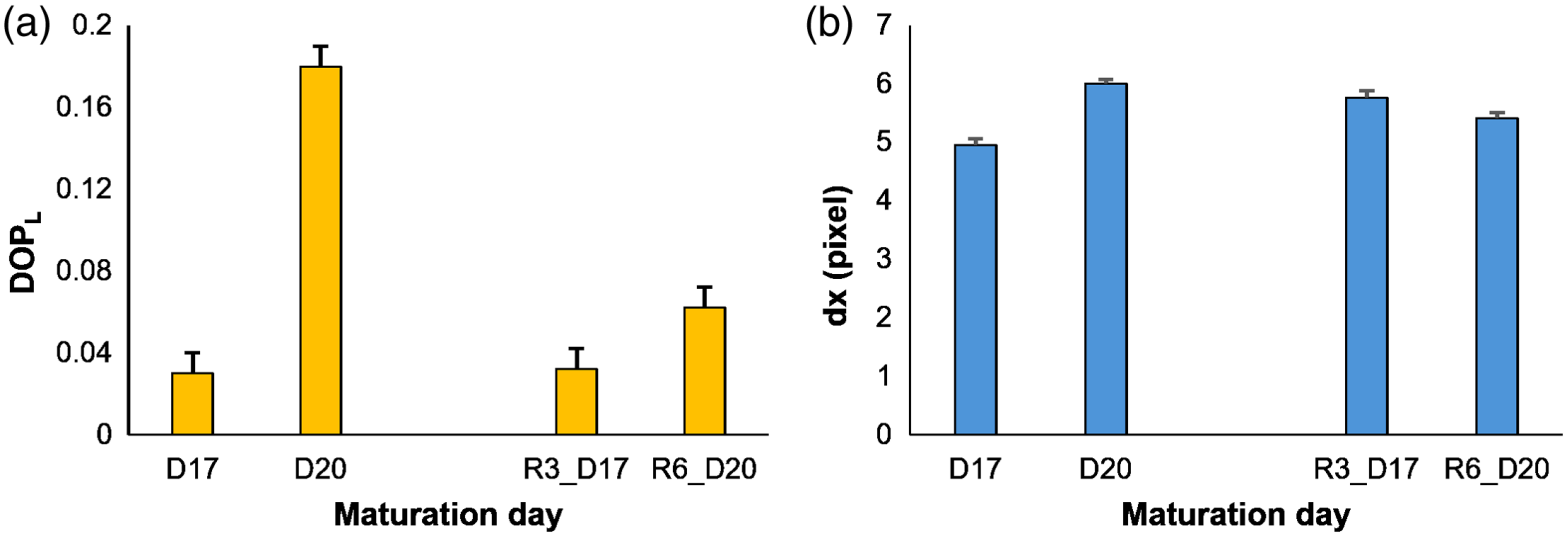
Comparison of speckle parameters: (a) ${\mathrm{DOP}}_{L}$ and (b) dx between standard controls and groups with repeated incubator removals.

As illustrated in Fig. 3, a change in speckle parameters responses was evident between the standard RHE controls and the control groups subjected to repeated removal from the incubator. The continuous increase in ${\mathrm{DOP}}_{L}$ [Fig. 3(a)] between days 17 and 20 of maturation for both control groups indicates an increase in the concentration of large scatterers with respect to the optical wavelength.^24^^,^^33^ However, the increase in the standard control group was more pronounced. Simultaneously, the dx value increased between days 17 and 20 of maturation whereas the dx value decreased between R3_D17 and R6_D20 samples [Fig. 3(b)], suggesting changes in RHE structure. These variations were likely attributed to external factors introduced during the RHE removal from the incubator. As a matter of fact, the controlled environment of the incubator provides optimal conditions for cell growth, including temperature, humidity, and ${\mathrm{CO}}_{2}$ levels. Disrupting this environment can lead to stress, altered metabolic processes, and impaired cellular functions. In fact, temperature fluctuations can affect cell metabolism and growth rates.^35^ Although heat stress can cause inhibited cellular processes and protein damage, cold stress can lead to changes in lipid metabolism and membrane fluidity. Both types of stress can activate common stress response pathways and cause oxidative stress. The specific responses to temperature stress can vary depending on the cell type, duration, and intensity of the stress.^36^ In addition, changes in the microenvironment, such as variations in oxygen levels,^37^ pH, and ${\mathrm{CO}}_{2}$ concentrations,^38^ can influence cell differentiation and biosynthesis. The interplay between temperature and environmental factors can have complex effects on cell culture outcomes, as temperature changes can affect the pH of the media due to altered ${\mathrm{CO}}_{2}$ solubility.^38^

To mitigate the effects of all these factors, we employed a relative comparison while assessing the blue light impact on RHE with respect to control groups. For each irradiation condition, we compared the irradiated samples to a corresponding control that had been simultaneously removed from the incubator. This ensured that both the irradiated and control samples experienced the same experimental perturbations, allowing us to evaluate only the contribution of irradiation.

### 3.2 Effect of Blue Light Irradiation on Barrier Function

**Fig. 4:**
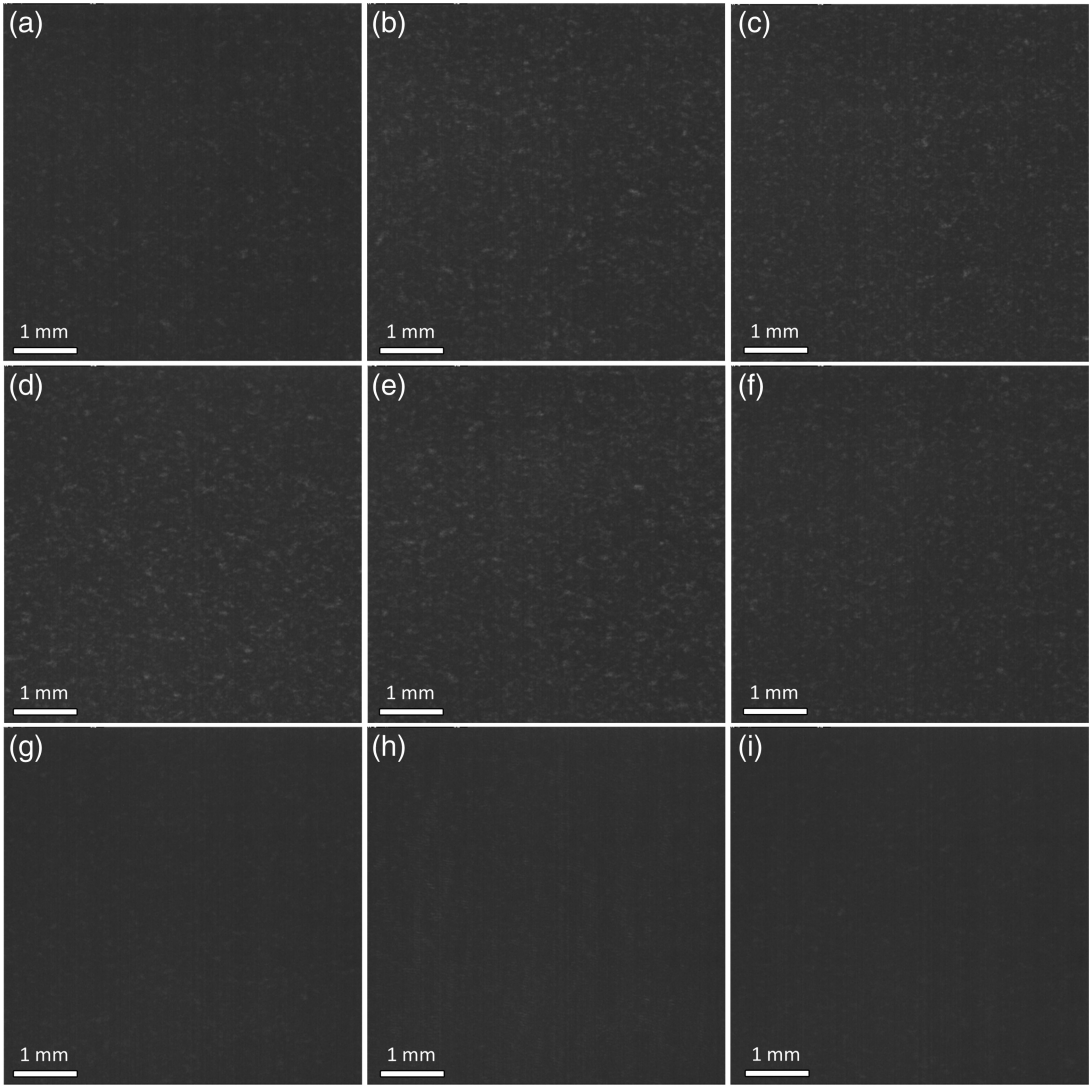
Speckles image recorded using linear parallel polarized detection of the RHE: Control samples at (a) R3_D17, (d) R4_D18, and (g) R6_D20; samples irradiated with 415 nm at (b) R3_D17, (e) R4_D18, and (h) R6_D20; and samples irradiated with 455 nm at (c) R3_D17, (f) R4_D18, and (i) R6_D20.

This study examined the impact of varying blue light doses on skin barrier function using RHE at two wavelengths: 415 and 455 nm. The effects of varying irradiation repetition (3, 4, and 6 times), total dose (150, 200, and $300\;J/{\mathrm{cm}}^{2}$), and RHE maturation day (17, 18, and 20) were studied. Various speckle (Fig. 4) parameters were employed to assess skin barrier integrity. For irradiated samples, the observed variations are affected by both the maturation stage and the irradiation dose. To emphasize the effects of irradiation, results are expressed as ratios relative to their respective control samples from the same maturation day.

#### 3.2.1 Impact of blue light irradiation on the degree of polarization

To comprehensively assess the scattering properties of RHE and identify the predominant type of backscattered photons throughout the blue light irradiation process, we measured the degree of linear polarization.

**Fig. 5:**
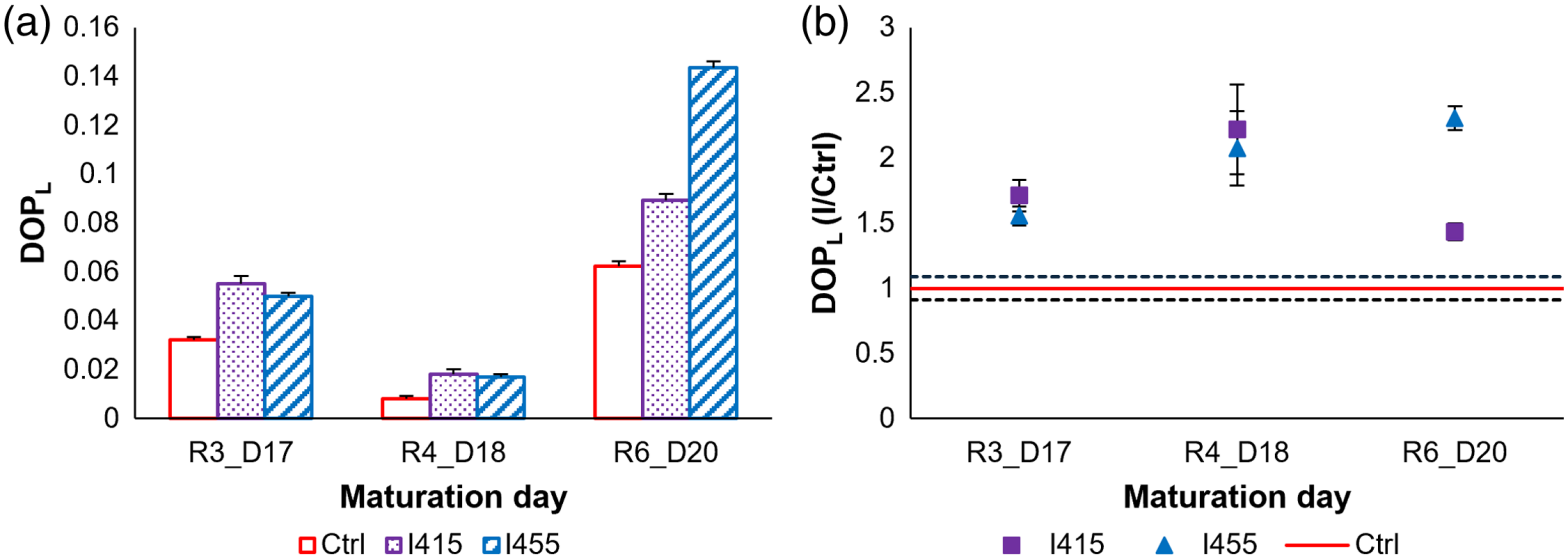
Variation of the ${\mathrm{DOP}}_{L}$ for RHE samples, among R3_D17, R4_D18, and R6_D20, in both (a) absolute values and (b) relative values calculated as a ratio of each irradiated sample (I) to its corresponding control (Ctrl).

Data presented in Fig. 5 reveal significant variations in ${\mathrm{DOP}}_{L}$ values for RHE samples across different maturation days and irradiation treatments.

Figure 5(a) shows a general trend of higher ${\mathrm{DOP}}_{L}$ values for irradiated samples compared with the control in all maturation phases (R3_D17, R4_D18, and R6_D20) for both wavelengths. Between R3_D17 and R4_D18, a decrease in ${\mathrm{DOP}}_{L}$ values was noted for all samples followed by a marked increase for R6_D20. Among all samples, the 455 nm irradiation consistently yielded the highest ${\mathrm{DOP}}_{L}$ values, particularly at R6_D20, reaching levels comparable to the standard control samples that were kept in the incubator. This aligns with previous research suggesting that moderate blue light exposure can be beneficial.^7^ Although exposure to 415 nm showed an upward trend in ${\mathrm{DOP}}_{L}$ values, they were slightly lower when compared with the values of standard control samples kept in the incubator.

When considering relative differences [Fig. 5(b)], the variation between irradiated samples and the control widened between R3_D17 and R4_D18, for both wavelengths. As previously described, control group values were normalized to 1 to facilitate the relative comparison of each irradiated sample with its corresponding control. A confidence interval is defined around 1 using the standard error of the mean of the control group. Variations where data points remain within this interval are not considered significant. Although this variation decreased for 415 nm irradiation between R4_D18 and R6_D20, it continued to increase for 455 nm irradiation, suggesting a more sustained effect at this wavelength.

#### 3.2.2 Impact of blue light irradiation on speckle grain size

**Fig. 6:**
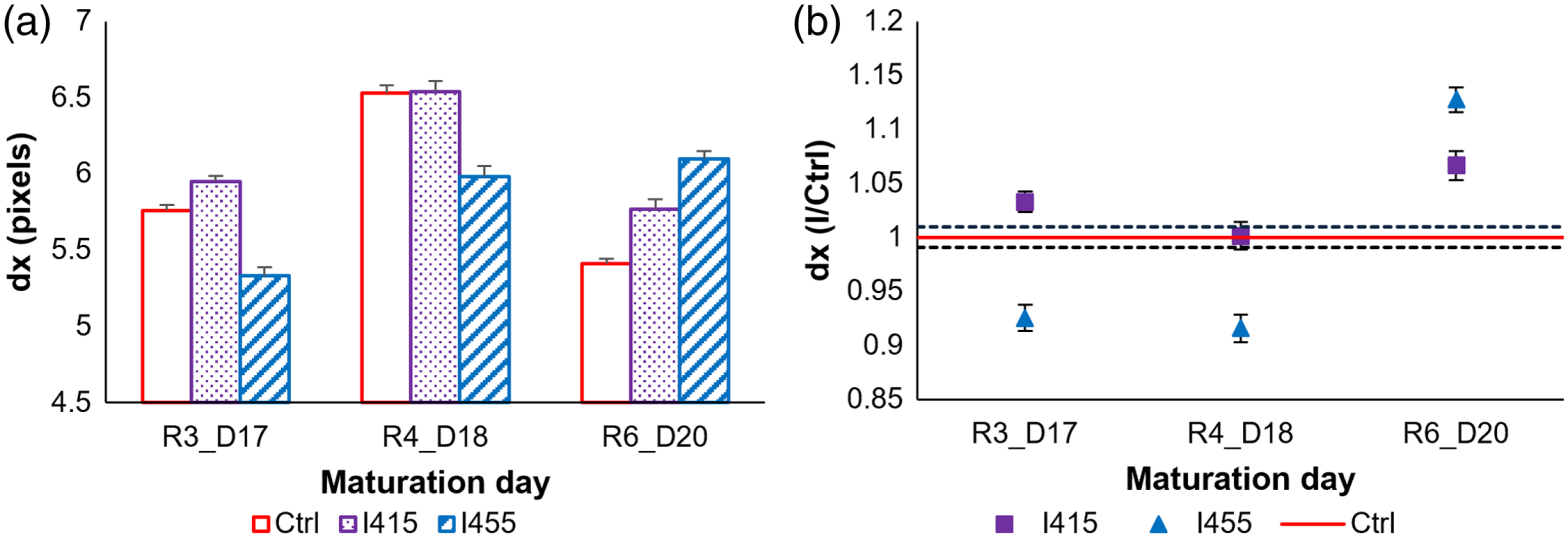
Variation of the horizontal speckle grain size for RHE samples, among R3_D17, R4_D18, and R6_D20, in both (a) absolute values and (b) relative values calculated as a ratio of each irradiated sample (I) to its corresponding control (Ctrl).

Figure 6 shows the evolution of horizontal speckle grain size (dx) for both irradiated and control RHE samples. A comparison of the irradiated samples (I415 and I455) to the control (Ctrl) reveals that blue light irradiation can significantly alter the horizontal speckle grain size of RHE.

For R3_D17, compared with the control group, the 415 nm irradiated samples exhibited a slight increase in dx, whereas for 455 nm, a marked decrease was observed.

At D18, all RHE samples showed an increase in the horizontal speckle grain size compared with D17. The relative dx values revealed no significant effect of the 415 nm irradiations [Fig. 6(b)]. The significantly lower relative values for the 455 nm samples are comparable to the observation at D17. It is worth noticing that the lowest energy radiation (455 nm) induced more significant modifications at both D17 and D18 when compared with the impact of I415. Moreover, the I455 obtained values are comparable to those observed with the standard control.

Compared with R4_D18, dx of both 415 nm irradiated and control RHE samples decreased at R6_D20. However, this decrease is more marked for the control. Absolute and relative values for 415 nm were significantly higher.

Contrary to the other samples, those irradiated by 455 nm showed no decrease in the absolute values of the dx revealing a maintained grain size at the surface. These values are significantly higher compared with the control. Interestingly, and as noticed for D17, 455 nm irradiation samples exhibited similar values to the standard control.

## 4 Discussion

The observed increase in ${\mathrm{DOP}}_{L}$ values is primarily attributed to a higher concentration of large scatterers with respect to the optical wavelength within the diffusing medium.^24^ In our study, these scatterers are likely to be corneocytes that generally measure about 30 mm in diameter and can have a thickness of 0.5 to 0.8 mm.^39^ This suggests that the irradiation might have indirectly influenced corneocyte concentration or their organization within the stratum corneum.

The observed variations in dx suggest that blue light irradiation can intervene in the skin barrier structure. An increase in dx indicates that a surface backscattering predominantly occurs, suggesting that RHE has a rougher surface and potentially a less organized skin barrier. On the opposite, a decrease in dx with larger scattering spots indicates that light is interacting with deeper and more organized structures within the RHE, and hence, a volume backscattering is predominant. When cells are densely packed and exhibit a more uniform orientation, incident photons tend to penetrate further in the RHE, interacting with the internal structure rather than the surface.

These speckle imaging findings align with our complementary molecular analyses, which revealed significant wavelength-dependent effects of blue light exposure on the skin barrier. Irradiation led to changes in protein secondary structures and lipid organization, as well as modifications in the conformation and composition of epidermal lipids, with distinct impacts between 415 and 455 nm. Regarding protein structures, exposure to 455 nm light resulted in delayed effects compared with 415 nm, with noticeable changes emerging at higher cumulative doses. Similarly, for lipid composition, 455 nm required greater cumulative exposure to induce lipid alterations comparable to those observed with 415 nm.^28^

The wavelength-dependent effects observed in both our previous findings and this study highlight the importance of considering the specific wavelength of blue light exposure when evaluating its impact on skin health. These results align with prior research highlighting the distinct impacts of different light spectra on skin cells.^40^

The complementary nature of these findings enhances our understanding of how blue light exposure influences skin barrier structure and function at both macroscopic and molecular scales.

## 5 Conclusion

Our findings suggest that blue light irradiation can significantly influence the structural properties of RHE. The observed changes in speckle parameters, including the degree of linear polarization (${\mathrm{DOP}}_{L}$) and horizontal speckle grain size (dx), indicate alterations in the skin barrier’s scattering properties in terms of concentration of large scatterers with respect to the optical wavelength and RHE organization, respectively.

The dependence of the response to irradiation on RHE maturation day suggests that the skin’s sensitivity to blue light may vary at different stages of maturation or aging.

The 455 nm wavelength seems to compensate for the disruptions observed in the control samples. This confirms that the balance between beneficial and detrimental effects is both wavelength- and dose-dependent.

Although speckle imaging did not provide specific information on the composition of RHE samples at each stage of their maturation or their exposure to blue light, this imaging technique allows describing, at a macroscopic level, structural changes that reflect microscopic alterations. Further research is needed to understand the underlying mechanisms by which blue light irradiation affects skin barrier structure and to assess the long-term consequences of such exposure.

## Data Availability

The data that support the findings of this article can be requested from the authors upon reasonable request.
